# Comparison of Microleakage of Mineral Trioxide Aggregate Apical Plug Applied by the Manual Technique and Indirect Use of Ultrasonic with Different Powers

**DOI:** 10.30476/DENTJODS.2020.85876.1157

**Published:** 2021-12

**Authors:** Mamak Adel, Zahra Salmani, Navid Youssefi, Behrouz Heidari

**Affiliations:** 1 Dept. of Endodontics, Dental Caries Prevention Research Center, Qazvin University of Medical Sciences, Qazvin, Iran; 2 Dept. of Periodontics, Faculty of Dentistry, Alborz University of Medical Sciences, Karaj, Iran; 3 Postgraduate Student of Prosthodontics, Student Research Committee, Qazvin University of Medical Sciences, Qazvin, Iran; 4 Postgraduate Student of Endodontics, Student Research Committee, Qazvin University of Medical Sciences, Qazvin, Iran

**Keywords:** Mineral Trioxide Aggregate, Apexification, Ultrasonics, Dental Leakage

## Abstract

**Statement of the Problem:**

A mineral trioxide aggregate (MTA) apical plug is commonly applied prior to endodontic treatment of open-apex teeth. However, difficult application and condensation of MTA in the apical region is a drawback of this technique.

**Purpose:**

This study aimed to compare the microleakage of MTA apical plug applied by the manual technique and indirect use of ultrasonic with different powers.

**Materials and Method:**

In this *in vitro*, experimental study, divergent open apices were created in 48 single-rooted, single-canal teeth using ProFile. The teeth were randomly divided into four experimental groups (n=10). All groups received 5-mm thick MTA apical plug at the apical region using one of the following methods. In group 1, MTA was manually condensed while in groups 2-4, indirect ultrasonic energy with minimum, medium, and maximum power levels was used for MTA plug condensation. After setting of MTA, the apical microleakage of the MTA plug was quantified using the fluid filtration method. Data were analyzed using the Mann-Whitney and Kruskal-Wallis tests (*p*< 0.05).

**Results:**

Significant differences were noted in microleakage of MTA plug between the manual group and ultrasonic groups with medium (*p*= 0.043) and maximum (*p*= 0.029) power levels. No significant difference was noted in microleakage of other groups (*p*> 0.05).

**Conclusion:**

Considering the current results, it seems that application of MTA with indirect ultrasonic energy at medium or high power level would decrease the microleakage of MTA plug in open-apex root canals.

## Introduction

The process of dentin formation and root development stops when an immature tooth undergoes pulp necrosis. Resultantly, the root canal walls remain thin, and the apex remains open. In such cases, it is imperative to close the apex by artificially inducing the formation of a calcified barrier to allow condensation of root filling materials and improve apical seal. Inadequate apical seal is the most important reason for failure of non-surgical root canal treatment [ 1 ]. Apexification is a commonly used technique to induce the closure of apical foramen in necrotic immature teeth. Single-session apexification by use of an apical plug and formation of an artificial barrier is an alternative to multi-session apexification with calcium hydroxide. In this technique, biocompatible materials are used to create an apical seal and allow root canal filling [ 1 ]. 

Mineral trioxide aggregate (MTA) is among the dental materials proposed for this purpose. MTA has ideal properties such as optimal biocompatibility [ 2 ], bacteriostatic activity [ 3 ], favorable sealability of the root canal and perforation sites [ 4 ] and the ability to set even in presence of blood [ 5 ]. The success rate of endodontic treatment of open-apex teeth with a MTA apical plug is reportedly 95.5% [ 6 ]. However, difficult application and condensation of MTA in the apical region is a drawback of using MTA for apical plug. Evidence shows that the technique of application of MTA is highly important [ 6 - 7 ]. Indirect application of ultrasonic energy on an endodontic plugger has been recommended for MTA delivery into the root canal to enhance the condensation of MTA in the apical region for formation of an apical plug [ 7 - 9 ]. 

Information regarding an ideal method for delivery and application of MTA into the canal is limited and controversial. Some studies have demonstrated that application of ultrasonic energy during MTA application leads to void formation and results in a filling with lower density and homogeneity compared with manual condensation [ 10 - 11 ]. On the other hand, some other studies have shown that condensation of MTA using ultrasonic energy provides higher compressive strength and higher surface microhardness of MTA compared with manual condensation [ 9 , 12 ]. Another studies indicated that indirect application of ultrasonic energy would improve the adaptation of MTA to dentinal walls [ 13 - 14 ]. Basturk *et al*. [ 15 ] discussed that the compressive strength of MTA, applied by using ultrasonic energy, had no significant difference with that in manual application technique. 

Considering the existing controversy in the literature regarding the preferred technique of application of MTA, and the significance of treatment of open-apex teeth, this study aimed to compare the microleakage of MTA plug applied by the manual technique and indirect use of ultrasonic with different powers. 

## Materials and Method

This *in vitro*, experimental study evaluated 48 single-rooted, single-canal maxillary central incisors with closed apices and no caries, cracks or root curvature. The selected teeth had been extracted due to different reasons such as hopeless periodontal prognosis.

A total of 48 qualified teeth were randomly divided into four experimental (n=10) and four positive and negative control (n=2) groups. For the purpose of standardization, the teeth were decoronated at the cementoenamel junction using a diamond disc and high-speed hand-piece such that 13±1mm of root length remained. Next, 3mm of the root apex was cut perpendicular to the root by a straight carbide fissure bur (Teezkavan, Tehran, Iran) to eliminate the apical delta and standardize the canal apex (root length in all teeth was 10±1mm). The root canals were cleaned and shaped using Peeso reamers #1, #2 and #3 (Dentsply Maillefer, Switzerland) 1mm longer than the working length and Peeso reamer#4 to the working length. Divergent open apices were prepared as retrograde using ProFile (06/40; Dentsply Maillefer, Switzerland). For this purpose, the file was introduced into the canal as retrograde by 16 mm to enlarge the root-end to 1.36 mm diameter [ 7 ]. In order to ensure the correct diameter of the apical end, it was measured using a digital caliper (CD-6CS Digimatic Caliper; Mitutoyo Corp., Japan) with 0.01 mm accuracy. Sodium hypochlorite (5.25%) was used to rinse the canals during instrumentation. Moreover, 17% EDTA (Ethylenediaminetetraacetic acid) (MD Cleanser; Meta Co., Korea) gel was used for 3 minutes to eliminate the smear layer. The canals were then rinsed with 10 cc of distilled water. The canals were dried and the teeth were randomly divided into four experimental (n=10) and four positive and negative control (n=2) groups. Four teeth in the positive control group underwent root-end preparation as explained above, but did not receive the MTA plug. They were tested by the fluid filtration method. Of the four teeth in the negative control group, two received the apical plug with indirect ultrasonic technique and two received the apical plug by the manual technique. Eventually, the root surfaces were coated with wax. To enhance the application of MTA plug and to better simulate the periapical resistance present in the clinical setting, the teeth were mounted in holes created in a moist flower sponge. ProRoot MTA powder (Dentsply Maillefer, Switzerland) was mixed with sterile water in 3:1 ratio [ 16 ] and delivered into the canal using a MTA gun (Dentsply Maillefer, Switzerland), and condensed at the apical end with 5 mm thickness. 

In all groups, application and condensation of MTA plug were performed in three steps by application of 2-, 2- and 1-mm thick layers. In group 1, the MTA plug was condensed manually using an endodontic plugger. In group 2, after the application of each layer of MTA plug, it was condensed by indirect use of ultrasonic energy (Various750-NE134; NSK, Japan) at minimum power level by touching the ultrasonic tip (P10 tip) with the plugger shaft without contacting the tooth walls for 2 s [ 11 , 14 , 17 ]. In group 3, the MTA plug was condensed by indirect use of ultrasonic energy with the power level of 2.5 (medium) for 2 s. In group 4, the MTA plug was condensed by indirect use of ultrasonic energy at maximum power level for 2 s. It should be noted that the ultrasonic device had minimum, 1, 2, 3, 4, and maximum power levels. Excess MTA on the walls was removed using a #50 stainless steel K-file (Dentsply Maillefer, Switzerland). Immediately after placement of the MTA plug, a radiograph was obtained to ensure its adequate thickness, absence of voids, and optimal adaptation to the canal walls. Next, moist paper points #80 (Ariadent, Tehran, Iran) were placed in the canals. The samples were incubated at 37° and 100% humidity for 7 days. After setting of the MTA plug, the roots were removed from the sponge, and adequate hardness of MTA was ensured using a file. The amount of microleakage was quantified using a fluid filtration model.

The fluid filtration model used in this study was equipped with an air tank, which had a manometer for precise adjustment of pressure at 0.5 atmosphere. A specific plastic tube was connected to the air source, and its end part was connected to an Erlenmeyer flask. Two holes were made in the lid of the Erlenmeyer flask, one for the entrance of air and the other for immersion in fluid. A micropipette (0.1 cc) was fixed and its end was connected to a three-valve tube by a latex pipe (0.5 cm in diameter). The upper side of the three-valve tube was connected to a syringe, which was used to create air bubbles through the micropipette. The lower side was used to connect the specimens. Microleakage in each specimen was recorded in millimeters (mm) and converted to µL/min/cm H_2_O.

Normal distribution of data was evaluated using the Shapiro-Wilk test. Since the data were not normally distributed and considering the presence of four independent groups, the non-parametric Kruskal-Wallis test was applied to compare the groups regarding microleakage. The non-parametric Mann Whitney test was used for pairwise comparisons of the groups. *p*< 0.05 was considered statistically significant.

## Results

No microleakage was noted in the negative control group. Severe microleakage was noted through the apex of teeth in the positive control group. Table 1 shows the mean apical microleakage of the experimental groups. Comparison of microleakage of the groups showed that the amount of microleakage in group 3 (medium ultrasonic power) had a significant difference with that in group 1 (manual condensation) (*p*= 0.043). Furthermore, microleakage in group 4 (use of maximum ultrasonic power) had a significant difference with that in group 1 (manual condensation) (*p*= 0.029). No significant difference was noted in microleakage of other groups (different ultrasonic power levels with each other and minimum ultrasonic power and manual condensation) (*p*> 0.05). 

**Table 1:** Mean microleakage of the four experimental groups

Groups	Number	Mean microleakage (µL/min/cm H_2_O)	Std. deviation
Use of ultrasonic energy with minimum power	10	21.65	1.82
Use of ultrasonic energy with medium power	10	17.95	6.92
Use of ultrasonic energy with maximum power	10	15.30	5.85
Manual condensation	10	27.10	8.69

## Discussion

Provision of an apical barrier is imperative during cleaning and shaping of root canals to achieve optimal apical seal. In absence of an apical barrier, it would be difficult to prevent the apical extrusion of root filling material and achieve an apical seal. In cases with apical root resorption, apical perforation and necrotic immature teeth, an apical constriction does not exist. In such cases, in order to confine the root filling materials to the canal space, provision of an artificial apical barrier is imperative [ 18 ]. Several materials have been proposed for use as apical plug by the researchers such as calcium hydroxide [ 19 ], MTA [ 20 ] and dentin chips [ 21 ]. Of the suggested materials, MTA has a superior marginal adaptation and biocompatibility compared with other materials [ 22 ]. Considering the reverse funnel shape of open-apex teeth, we standardized the divergent apical foramen as retrograde using ProFile [40/6%] [ 23 ]. Controversy exists regarding the adequate thickness of MTA apical plug [ 7 , 23 - 24 ]. Nonetheless, most researchers have agreed on 5 mm thickness of apical plug [ 25 - 26 ]. Thus, MTA apical plug with 5 mm thickness was applied in this study. In addition, the teeth were mounted in a moist sponge during filling of open-apex roots according to a study by Martin *et al*. [ 23 ] in order to simulate the periapical tissue and prevent the apical extrusion of root filling material. Considering the previous observations [ 7 , 25 ], we indirectly transferred the ultrasonic energy to an endodontic plugger for the condensation of MTA plug. 

Aminoshariae *et al*. [ 8 ] showed that direct application of ultrasonic energy for condensation of MTA plug, compared with manual condensation by a plugger, increased voids and decreased the adaptation of root filling materials to the canal walls. In this study, ultrasonic energy was indirectly transferred to a plugger after eliminating the smear layer by using EDTA. Araujo *et al*. [ 27 ] demonstrated that indirect application of ultrasonic energy (for 2 seconds) following smear layer removal significantly improved the marginal adaptation while use of ultrasonic energy without elimination of smear layer did not increase the marginal adaptation. 

Parashos *et al*. [ 17 ] showed that MTA condensation using indirect ultrasonic energy for 2 seconds decreased the microleakage, porosities, and voids compared with manual condensation. Thus, according to this study, increasing the duration of use of ultrasonic energy for more than 2 seconds for condensation of MTA plug would significantly increase the void formation and microleakage and decrease the microhardness of MTA plug. Previous studies on the efficacy of ultrasonic energy for MTA condensation have assessed properties such as compressive strength, flexural strength, marginal adaptation, density, and porosities [ 7 - 9 , 11 - 13 , 28 ]. Nonetheless, limited studies have evaluated the effect of ultrasonic on microleakage of MTA plug [ 7 , 17 ]. Previous studies used the dye penetration or bacterial leakage techniques for assessment of microleakage [ 14 , 25 ]. In addition, microleakage can be assessed using different techniques such as fluid filtration and electrochemical method [ 29 ]. The fluid filtration technique, employed in this study, is a reproducible quantitative method and does not destruct the tooth structure. This technique does not require a marker or an indicator and therefore, does not have their related complications (such as size or pH). The results are recorded automatically and are highly accurate. Very small values are also recorded [ 29 ]. 

It seems that the transfer of ultrasonic vibrations through the plugger to the MTA would eliminate the voids between the particles and result in formation of a denser and more homogenous MTA plug. On the other hand, it would minimize the gap between the MTA plug and root canal walls, which leads to eventual reduction in microleakage. Yeung *et al*. [ 30 ] showed that using ultrasonic energy for MTA condensation compared with the manual technique increased the weight of MTA mass. Escribano-Escriva *et al*. [ 14 ] indicated that indirect application of ultrasonic energy enhanced the adaptation of MTA plug to the canal walls. Araujo *et al*. [ 27 ] and Friedl *et al*. [ 28 ] stated that ultrasonic energy improved the marginal adaptation of MTA plug to the canal walls and increased its density. Lawley *et al*. [ 7 ] assessed the apical seal provided by the MTA plug after its manual application and by the use of ultrasonic energy. They showed that indirect application of ultrasonic energy with 50% power decreased the apical microleakage after the placement of MTA plug. We used minimum, medium, and maximum power levels of ultrasonic device. Although use of medium and high ultrasonic power levels for MTA plug application decreased the microleakage compared with the manual technique (*p*= 0.043 and *p*= 0.029, respectively), use of ultrasonic energy with minimum power level did not decrease the microleakage compared with the manual technique (*p*> 0.05). Thus, it seems that minimum ultrasonic power level is not sufficient for achieving a homogenous mass with maximum adaptation to the canal walls, and higher power levels (medium and high) are required for this purpose. 

Basturk *et al*. [ 15 ] used the power level 5 of ultrasonic energy for MTA condensation and measured its compressive strength compared with the compressive strength of manually condensed MTA plug. They showed that the compressive strength of MTA applied by the use of ultrasonic energy was significantly higher than that applied manually. In the study by Basturk *et al*. [ 15 ], indirect use of ultrasonic energy decreased the porosities and increased the compressive strength of MTA plug compared with the manual technique. However, this difference was not significant. The difference between the results yielded by their study and ours can be due to the use of an impression to fabricate the MTA plug instead of root canal and long duration of using ultrasonic energy (30 s) in their study. However, Yeung *et al*. [ 30 ] used minimum power level of ultrasonic energy for condensation of MTA plug and concluded that the weight of MTA mass significantly increased when ultrasonic was used compared with that when the manual technique was employed. Their results were different from ours, which may be due to the absence of standardization and absence of any information on minimum and maximum power levels of ultrasonic device. El-Ma'aita *et al*. [ 10 ] stated that use of manual technique resulted in a denser filling compared with the ultrasonic method. In addition, Ghasemi *et al*. [ 11 ] reported that use of manual technique decreased the number of voids in the apical plug [ 11 ]. Aminoshera *et al*. [ 8 ] indicated that manual application resulted in superior homogeneity and fewer voids compared with the ultrasonic technique [ 8 ]. The controversy between their results and ours can be due to the direct use of ultrasonic energy for a longer period of time (30 s) in their studies, which might result in formation of air bubbles in the MTA mass and reduction in density compared with the use of manual technique for MTA condensation.

## Conclusion

Considering the current results, it appears that application of MTA with indirect ultrasonic energy at medium or high power level would decrease the microleakage of MTA plug in open-apex root canals. However, considering the results of this study and differences between our methodology and that of previous studies, it appears that determining the more appropriate method for MTA plug application in the clinical setting requires further studies and standardization. 

## Conflict of Interest

The authors have no conflicts of interest to disclose.

## References

[1] Morse DR, O'Larnic J, Yesilsoy C ( 1990). Apexification: review of the literature. Quintessence Int.

[2] Koh ET, McDonald F, Pitt Ford  TR, Torabinejad M ( 1998). Cellular response to Mineral Trioxide Aggregate. J Endod.

[3] Torabinejad M, Hong CU, Pitt Ford  TR, Kettering JD ( 1995). Antibacterial effects of some root end filling materials. J Endod.

[4] Arens DE, Torabinejad M ( 1996). Repair of furcal perforations with mineral trioxide aggregate: two case reports. Oral Surg Oral Med Oral Pathol Oral Radiol Endod.

[5] Torabinejad M, Higa RK, McKendry DJ, Pitt Ford  TR ( 1994). Dye leakage of four root end filling materials: effects of blood contamination. J Endod.

[6] Moore A, Howley MF, O'Connell AC ( 2011). Treatment of open apex teeth using two types of white mineral trioxide aggregate after initial dressing with calcium hydroxide in children. Dent Traumatol.

[7] Lawley GR, Schindler WG, Walker WA, Kolodrubetz D ( 2004). Evaluation of ultrasonically placed MTA and fracture resistance with intracanal composite resin in a model of apexification. J Endod.

[8] Aminoshariae A, Hartwell GR, Moon PC ( 2003). Placement of mineral trioxide aggregate using two different techniques. J Endod.

[9] Basturk FB, Nekoofar MH, Günday M, Dummer PM ( 2013). The effect of various mixing and placement techniques on the compressive strength of mineral trioxide aggregate. J Endod.

[10] El-Ma'aita AM, Qualtrough AJ, Watts DC ( 2012). A micro-computed tomography evaluation of mineral trioxide aggregate root canal fillings. J Endod.

[11] Ghasemi N, Janani M, Razi T, Atharmoghaddam F ( 2017). Effect of different mixing and placement methods on the quality of MTA apical plug in simulated apexification model. J Clin Exp Dent.

[12] Nekoofar MH, Aseeley Z, Dummer PM ( 2010). The effect of various mixing techniques on the surface microhardness of mineral trioxide aggregate. Int Endod J.

[13] Sisli SN, Ozbas H ( 2017). Comparative Micro-computed Tomographic Evaluation of the Sealing Quality of ProRoot MTA and MTA Angelus Apical Plugs Placed with Various Techniques. J Endod.

[14] Escribano-Escrivá B, Micó-Muñoz P, Manzano-Saiz A, Giner-Lluesma T, Collado-Castellanos N, Muwaquet-Rodríguez S ( 2016). MTA apical barrier: In vitro study of the use of ultrasonic vibration. J Clin Exp Dent.

[15] Basturk FB, Nekoofar MH, Gunday M, Dummer PM ( 2015). Effect of varying water-to-powder ratios and ultrasonic placement on the compressive strength of mineral trioxide aggregate. J Endod.

[16] Parirokh M, Torabinejad M ( 2010). Mineral trioxide aggregate: a comprehensive literature review--Part I: chemical, physical, and antibacterial properties. J Endod.

[17] Parashos P, Phoon A, Sathorn C ( 2014). Effect of ultrasonicationon physical properties of mineral trioxide aggregate. Biomed Res int.

[18] Matt GD, Thorpe JR, Strother JM, McClanahan SB ( 2004). Comparative study of white and gray mineral trioxide aggregate [MTA] simulating a one- or two-step apical barrier technique. J Endod.

[19] Weisenseel JA  Jr, Hicks ML, Pelleu GB  Jr ( 1987). Calcium hydroxide as an apical barrier. J Endod.

[20] Torabinejad M, Chivian N ( 1999). Clinical applications of mineral trioxide aggregate. J Endod.

[21] Rossmeisl R, Reader A, Melfi R, Marquard J ( 1982). A study of freeze-dried [lyophilized] dentin used as an apical barrier in adult monkey teeth. Oral Surg Oral Med Oral Pathol.

[22] Maltezos C, Glickman GN, Ezzo P, He J ( 2006). Comparison of the sealing of Resilon, Pro Root MTA, and Super-EBA as root-end filling materials: a bacterial leakage study. J Endod.

[23] Martin RL, Monticelli F, Brackett WW, Loushine RJ, Rockman RA, Ferrari M, et al ( 2007). Sealing properties of mineral trioxide aggregate orthograde apical plugs and root fillings in an in vitro apexification model. J Endod.

[24] De-Deus G, Audi C, Murad C, Fidel S, Fidel R ( 2008). Similar expression of through-and-through fluid movement along orthograde apical plugs of MTA Bio and white Portland cement. Int Endod J.

[25] Kim US, Shin SJ, Chang SW, Yoo HM, Oh TS, Park DS ( 2009). In vitro evaluation of bacterial leakage resistance of an ultrasonically placed mineral trioxide aggregate orthograde apical plug in teeth with wide open apexes: a preliminary study. Oral Surg Oral Med Oral Pathol Oral Radiol Endod.

[26] Sisli SN, Ozbas  H ( 2017). Comparative Micro–computed Tomographic Evaluation of the Sealing Quality of ProRoot MTA and MTA Angelus Apical Plugs Placed with Various Techniques. J Endod.

[27] Araújo AC, Nunes E, Fonseca AA, Cortes MI, Horta MC, Silveira FF ( 2013). Influence of smear layer removal and application mode of MTA on the marginal adaptation in immature teeth: a SEM analysis. Dent Traumatol.

[28] Friedl CC, Williamson AE, Dawson DV, Gomez MR, Liu W ( 2016). Comparison of Mechanical and Indirect Ultrasonic Placement Technique on Mineral Trioxide Aggregate Retrofill Density in Simulated Root-end Surgery. J Endod.

[29] Veríssimo DM, do Vale  MS ( 2006). Methodologies for assessment of apical and coronal leakage of endodontic filling materials: a critical review. J Oral Sci.

[30] Yeung P, Liewehr FR, Moon PC ( 2006). A quantitative comparison of the fill density of MTA produced by two placement techniques. J Endod.

